# Supplementary figures and images for: An Early Block in the Replication of the Atypical Bluetongue Virus Serotype 26 in Culicoides Cells Is Determined by Its Capsid Proteins
Source: Viruses. 2021 May 15;13(5):919. doi: 10.3390/v13050919 (PMC8156691; doi:10.3390/v13050919)

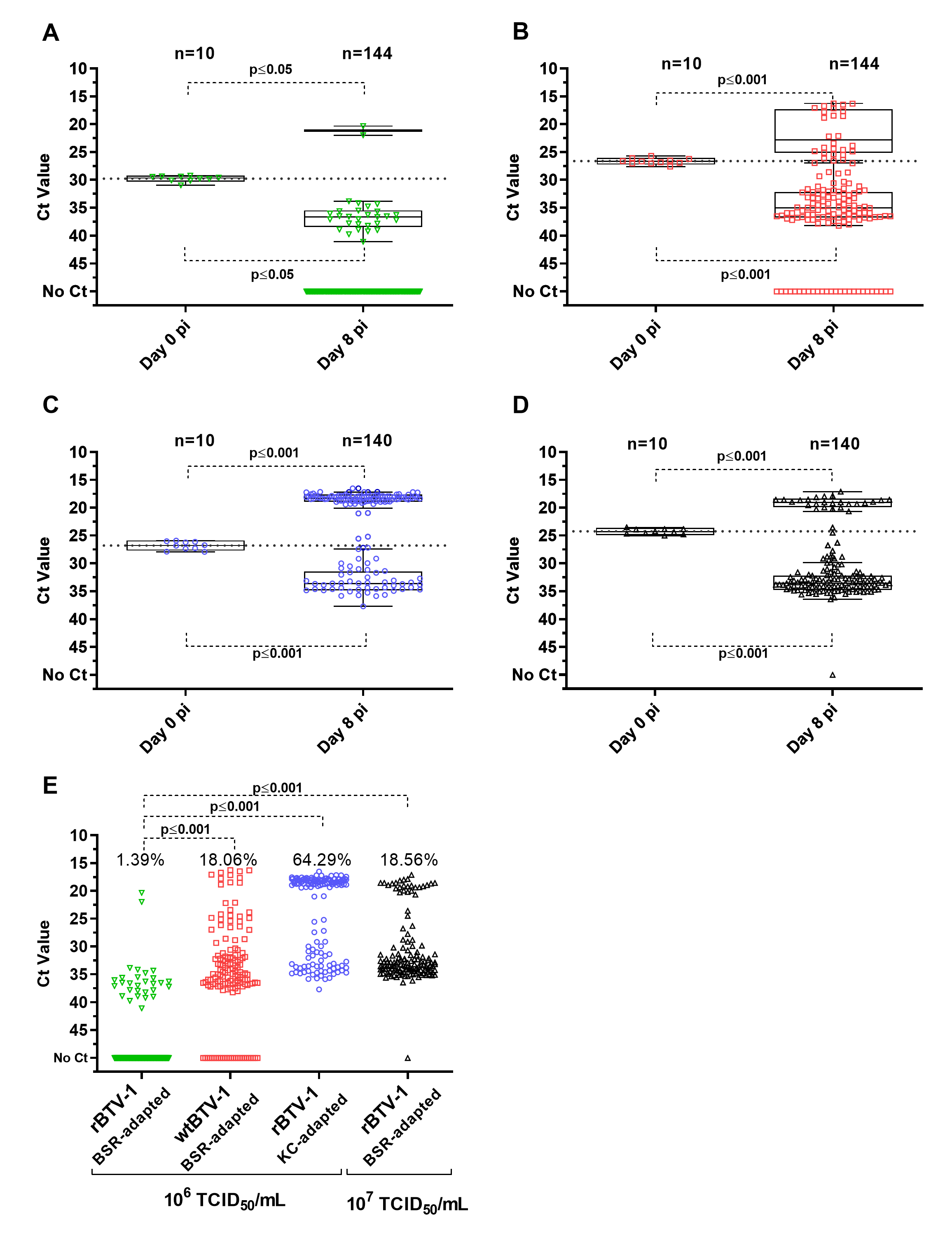

Supplement: Supplementary file 1 [file viruses-13-00919-s001.zip › viruses-1178162-supplementary.tif]
